# Health instruction in Nigerian schools: what are the missing links?

**DOI:** 10.11604/pamj.2014.19.360.4587

**Published:** 2014-12-09

**Authors:** Oladele Simeon Olatunya, Saheed Babajide Oseni, Oyeku Akibu Oyelami, Caleb Adegbenro, Nwadiuto Akani

**Affiliations:** 1 Paediatrics Department, Ekiti State University Ado Ekiti, Nigeria; 2 Paediatrics Department, Obafemi Awolowo University Ile-Ife, Nigeria; 3 Community Health Department, Obafemi Awolowo University Ile-Ife, Nigeria; 4 Paediatrics Department, University of Port Harcourt, Nigeria

**Keywords:** School Health Instruction, School children, Child health, Nigeria

## Abstract

**Introduction:**

School health instruction (SHI) is the instructional aspects of school health programme. It provides information on key health issues to school children who are in their formative years.

**Methods:**

A cross sectional descriptive study of all the primary schools in a focal Local Government Area in Nigeria was carried out to ascertain the implementation of SHI with regards to the contents, methods of delivery and teachers preparation for health teaching using an evaluation checklist for SHI.

**Results:**

There were more female pupils enrolled in the study area compared to their male counterparts with a male to female ratio of 0.9:1.0 and only 3.0% of the teachers had In-service training on health related issues in the previous five years preceding the study. 79.4% of the teachers had the recommended qualification to work in the schools. Teachings on emotional health, communicable diseases and safety education were sparingly given by 1.6%, 4.7% and 56% schools respectively. Only three (4.7%) schools (all private) had health instruction given by designated health education staff. No school gave health instruction at least thrice a week as recommended.

**Conclusion:**

Compliance with the implementation of SHI was very poor in the study area.

## Introduction

School Health Instruction (SHI) represents the instructional aspect of School Health Programme and it involves well planned and organized learning experiences for the school children under the guidance and supervision of teachers or accredited health personnel [1–3]. However, it‘s effectiveness depends on a number of factors such as: availability of instructional materials, skills and motivation of the instructor who may be the teacher or certified health personnel, use of appropriate teaching technique and the quality of the contents of the health instruction [1–4]. The target of primary schools for effective SHI delivery is based on the fact that, primary school children are in their formative years and are likely to easily imbibe any health lessons learnt in such a way that their lives will be affected positively as they grow to become adults [1–5].

In developing countries like Nigeria, there are multiple contenders for the often limited and ill appropriated resources [6]. The citizens of such countries will be better served if their SHI is effective bearing in mind the overwhelming evidence in support of the cost effectiveness of SHI as a veritable tool to turn around the health status of the citizens of a country [7]. While there are well entrenched instruments to monitor the implementation of SHI in developed countries [8], there is scanty literature in Nigeria on SHI with a few available directed towards evaluating the implementation of only parts of SHI effectiveness determinants and some of them are quite dated [9–11]. We assessed the compliance with the implementation of SHI in the study area with respect to the availability of all the key areas identified as determinants of its effectiveness with the aim of identifying areas of weaknesses and strengths, proffering appropriate recommendations to authorities concern with running the education sector in the country and add to the literature on SHI in Nigeria.

## Methods

This study was conducted among primary schools in Ilesa East Local Government Area (IELGA) of Osun State Nigeria. The Local Government Area (LGA) is populated mainly by The Yorubas of the south western part of Nigeria. It has a population of 105,416 [12]. The LGA has two higher institutions namely: The Osun State College of Education and The School of Health Technology. It also has a tertiary health facility: The Wesley Guild Hospital-a unit of The Obafemi Awolowo University Teaching Hospitals Complex Ile Ife. The College of Education trains middle cadre teachers required in primary schools in Nigeria. An approval was obtained from the Local Government School Education Authority before the study commenced. The consent and cooperation of each school head was sought by explaining the purpose and benefits of the research work to them in clear terms. Verbal consent was obtained from each participant. There were no reported adverse effects on learning.

The study was a school-based cross sectional descriptive type conducted between September 2010 and March 2011. It involved all primary schools (private and public) in Ilesa East Local Government Area of Osun State. Consideration was given to the timing and the conduct of the study so that normal teaching periods were not interrupted. An adapted school health instruction evaluation observational checklist Appedix I [1] was used to record information and score each school on their implementation of SHI. The checklist was adapted from “School Health Practice” by Anderson and Creswel [13].

The checklist have sections on the school administration data, availability of the various components of SHI such as the scope and contents of SHI delivered in the schools as obtainable in the National primary schools‘ curriculum [14], time allotted to health teaching, methods used in giving health instruction, teachers preparation for health instruction delivery during their trainings, availability of instructional materials, in-service trainings for teachers and graded health instruction for all grade levels in the schools. The checklist was administered by the researchers and a ‘face to face‘ interview conducted separately with the head teachers, two pupils selected randomly from each grade level and teachers involved in the delivery of health instruction in each school visited. The teachers‘ lessons preparation notes were checked to identify the scope and the contents of their instructions to the pupils while the two pupils had the contents of their notebooks scrutinized in order to corroborate or dispel their teachers‘ claims on SHI delivery. The head teachers provided information about the administrative data of the schools and other information on SHI that could not be directly observed by the researchers.

The teachers in each school were also asked questions about the scope of their trainings on heath instruction while the pupils provided information to either corroborate or dispel any claim of the teachers on any aspect of the checklist that could not be directly observed. For example, the lessons‘ timetable was not solely used to arrive at the frequency of health instruction delivery in each school this was further ascertained by asking the pupils how frequently this was done and corroborating their responses with findings in their notebooks. In order to enhance objectivity in the assessment, separate interviews were conducted with the pupils and the teachers in each school. According to the checklist, the maximum attainable and minimum acceptable scores on SHI were 41 and 27 respectively. Details of the scores for the various aspects of SHI under consideration are as shown in the observational checklist (Appendix I). A pilot study was carried out using two primary schools (one private and the other public) in Ilesa West Local Government Area (a location outside but close to the study area). The information obtained from the pilot study was used to modify the face to face interview with teachers and pupils to make data collection better.

The data obtained was entered into personal computer and analysed with the Statistical Programme for Social Science (SPSS) software version 16. Descriptive analysis was done using simple proportions and Means, standard deviations, percentages were determined as applicable. The results obtained from the public schools were compared with those of the private schools using the independent sample t-test and the Pearson's chi-squared (x^2^) tests as applicable. P values less than 0.05 were accepted as statistically significant.

## Results

### School administrative data

There were 64 primary schools consisting of 34 public and 30 private schools in Ilesa East Local Government Area with a total of 18,694 pupils in all the schools out of which 63.6% and 36.4% of them were in the public and private schools respectively. They had 9,140 male and 9,554 female pupils in all with an average male pupil to female pupil ratio of 1: 1.04. There were 912 teachers and the ratio of teachers to pupils was 1:20.5 (Table 1). Parents’ Teachers’ Associations (PTA) were present in 53 (82.8%) schools. As shown in Table 2, the highest academic qualification obtained by the teachers was Bachelor degree, while Secondary school certificate was the least qualification encountered. The commonest qualification encountered was the National Certificate on Education (NCE) and this was found among 76.3% of the teachers. All teachers (100%) in the public schools had the recommended academic qualifications to work in the schools compared to the (56%) recorded by their counterparts in the private schools.

**Table 1:** The distribution of pupils and teachers in the schools

Type of school	No of pupils	No of males	No of females	No of teachers	Teachers-Pupils ratio	Male-Female pupils ratio
Public (n = 34)	11894	5742	6152	568	1:21	0.9:1
Private (n = 30)	6800	3398	3402	344	1:20	1:1
Total (N = 64)	18694	9140	9554	912	1:20.5	1:1.04

**Table 2:** Distribution of teachers and their qualifications

Qualification	Publicn = 484n(%)	Privaten =428n(%)	TotalN= 912N(%)
*Bachelor of Education degree	28(6.0%)	0(0.0%)	28(3.1%)
Other Bachelor degree	0(0.0%)	100(23.3%)	100(11.0%)
*NCE	456(94.0%)	240(56.1%)	696(76.3%)
HND	0(0.0%)	42(9.8%)	42(4.6%)
OND	0(0.0%)	21(4.9%)	21(2.3%)
Grade II SSCE	0(0.0%) 0(0.0%)	14(3.3%) 11(2.6%)	14(1.5%) 11(1.2%)
	484(100.0%)	240(56.0%)	724(79.4%)

* Total no of teachers with Recommended qualifications

NCE & University degrees in education related fields are the recommended qualifications for primary school teachers in Nigeria [15]

KEY: NCE – National Certificate on Education

HND – Higher National Diploma

OND – Ordinary National Diploma

Grade II- Grade II Teachers Certificate

SSCE – Secondary School Certificate

### Time allocated to health instruction teaching

Health instructions were taught in hierarchical form in all (100%) schools, 43(67.2%) of them did so once a week (85.3% public vs 50% private schools) and 21(32.8%) twice a week (14.7% public vs 50% private schools). None of the schools gave health instruction thrice a week. The health instruction lesson periods were merged with physical education as a joint lesson period of between 45minutes to an hour in all schools and these were reflected thrice weekly in all the schools lessons‘ time table.

### Teachers’ preparation for health teaching

Six hundred and ten teachers (402 public vs 208 private) representing 92.7% of the 658 teachers (415 public vs 243 private) who responded to our questions on the assessment of their trainings in school while preparing for the teaching job claimed to have had any training on the components of SHI as available in the schools‘ curriculum. Only twenty (3.0%) teachers (16 public vs 4 private) had In-service training on health instruction in the past five years preceding this study.

### Content of health instruction in schools

As shown in Table 3, there was no significant difference between the two groups of schools with regards to the content of health instruction except in the aspect of HIV/AIDS where the public schools performed better (P = 0.021).

**Table 3:** Contents/ scope of health instruction in the schools

Contents /scope	Public N = 34 n	Private N = 30 n	Total N = 64 n (%)	χ^2^	P value
Growth and development	34	29	63 (98.4)	0.004	0.950
Personal health including hygiene, food and nutrition	34	28	62 (96.9)	0.656	0.418
Community health including communicable and non communicable diseases	1	2	3 (4.7)	*0.012	0.912
Drug abuse and emotional health	1	0	1 (1.6)	*0.000	1.000
Sex education &HIV/AIDS	30	18	48 (75.0)	5.354	0.021
Safety education including first aid and accident prevention	19	17	36(56.3)	0.004	0.950

* Chi-square (χ^2^) with Yate's correction applied

### Methods of health instruction

All the schools (100%) gave health instruction by integration with other subjects i.e. taught under different subjects such as in family living, social studies and physical education by teachers. Supplementary teaching aids and instructional materials like: chalkboards, audio-visual aids, illustration charts, pictures, posters, maps and textbooks were available in 38(59.4%) schools, 20 public (58.8%) vs 18 private(60.0%) majority of which were inadequate in quantity and quality. Four (6.3%) and ten (15.6%) schools had designated health instruction staffs/teachers and conducted health trips respectively. These were all private schools. No school had SHI given by medical specialists.

### Scores and their means comparison

No school attained the recommended minimum score of 27. The scores ranged from 14 to 22 for public schools and 12 to 25 for private schools out of a total obtainable score of 41 with means (SD) of 17.62 ±1.84 and 18.73 ±3.05 respectively. There was no significant difference in the mean scores of the two groups of schools (t = 1.795, df =62, p = 0.075).

## Discussion

Management of the education sector in Nigeria is on the concurrent list with the different levels of government sharing responsibilities [15]. The Federal Government provides the regulatory frameworks through bodies under her education ministry. The National Policy on Education (NPE) [15] spells out the minimum qualification and ratio of teachers to pupils expected in the primary school settings as well as how to improve the teachers‘ skills. The Universal Basic Education Commission (UBE) [16] serves to ensure that children of school going age are made available in schools to benefit from the activities of the NERDC and NPE. It can simply be regarded as the political arm for regulating the primary education programme in Nigeria [17].

In this study, there were more female pupils enrolled in the schools with a female to male ratio of 1.04:1. This is a shade better than the National average of 0.9:1 [18, 19] and does not indicate marginalization of the girl child with respect to educational opportunities in the study area. This may be a reflection that the inhabitants of the study area, particularly the girl child, are in tandem with the realization of the goals of the MDGS on equity, achieving universal education and other health related issues [20]. However, this finding may not be a true reflection of the girl child enrollment status in the whole state, the area of study being semi-urban with relatively more enlightened people.

The Teachers-Pupils ratio observed in this study falls within the recommended range [15] and is commendable. This could give room for effective delivery of SHI in view of the expected manageable size of pupils per teacher. Sadly, this was not the situation as none of the schools measure up to standard in the implementation of SHI [1]. A critical look at the distribution of the teachers shows that quite a number of them working in the private schools did not have the requisite qualifications. This may stem from the possibility that, some of them found their way into those schools out of frustration from the unemployment problems plaguing the country [6–19]. It might also be a manifestation of laxity in the country‘s education sector aided by lack of adequate supervision of teachers‘ recruitment. Inadequate quality control by the appropriate government agencies might have also aided inefficiency on their parts as many of them were not trained for teaching job primarily. That all the teachers in public schools had the requisite qualification required for teaching in the schools as recommended [15] is commendable. However, this did not impact positively on their performance in the implementation of SHI. Lack of motivation and commitment have been mentioned as possible reasons for the lack-luster performance of schools on the delivery of SHI and factors militating against the success of education programmes in Nigeria despite the seemingly adequate human resources [4, 17].

No school complied with the National Education Research Development Council's (NERDC) recommendation of not less than three times per week health teaching [14]. Of interest is the finding that whereas, in all the schools, the time table reflected three periods per week for Health and Physical education instruction, only one of such periods was used for teachings on health related issues in most schools while the remaining period(s) might have been used for physical exercises and other activities. This indicates that authorities in the schools perhaps know about the standard recommendation but choose not to adhere to it. It may be a manifestation of the laxity observed by Agusiegbe [9] that, trainee teachers may graduate without adequately covering the syllabus on health related teachings. Though physical exercise is good [21] having adequate health knowledge is equally good. Allocating separate periods to health teachings aside the physical education sessions may be the best way out rather than lump them together as this will prevent encroaching into their effective delivery.

Concerning the scope of health teaching, a high proportion of the schools were not teaching their pupils about safety and health education, control of communicable disease and HIV/AIDS despite their inclusion in the curriculum of health education in primary schools [14]. Safety and accident prevention lessons could help the pupils avert injuries given that they are playful, adventurous and as such, prone to injuries. Also, it could help enhance their safety in the wake of insecurity in Nigeria where school children are also victims [22]. That HIV/AIDS was less taught in the private schools despite its being a topical issue could be as a result of the presence of more unqualified teachers in these schools who are unlikely to realize the importance of such teaching or know the appropriate teaching techniques require at such elementary stage of learning that primary schools represent. Another interesting finding is that health instruction was being given with other subjects like physical education, family living and social studies. This may allow inadequate monitoring of the contents or scope of health instruction being given in these schools compared to that recommended in the schools’ curriculum [14]. This is in contrast to what obtained in developed countries where, there are well entrenched policies for monitoring effective delivery of health instruction in their schools [8].

The lack of designated health education staff in this study might have stemmed from the general beliefs that, teachers in Nigeria‘s primary schools are trained as generalists and as such, could teach any subject [14, 15]. This suggests that, health instruction in most of the schools was given by sundry instructors who may not have adequate knowledge on health issues or lack appropriate pre-employment training as observed with some of the teachers in the private schools. These observations might have contributed to the parlous state of SHI in the study area given the background that poor knowledge on health issues have been demonstrated among some primary school teachers in Nigeria [10, 11, 23, 24].

The relative lack of instructional materials in schools is not limited to this study. Authors from other parts of the country [4, 17, 18, 25, 26] have painted similar scenarios though to varying degrees. Presence of instructional materials like: chalkboards, audio-visual aids, illustration charts, pictures, posters, maps and textbooks not only make passage of health knowledge easy but also makes them last longer in the memory of pupils [2]. According to some authors [2, 27, 28], school children tend to remember better what they hear, see and act rather than mere hearing alone. The complete lack of visiting health personnel (who are more knowledgeable on health issues) to give health instruction in the schools could further worsen the situation given the lack of teaching aids. This contradicts the philosophy of primary health care in the country in which schools are targeted as one of the catchment areas for preventing childhood illnesses through appropriate SHP [1, 3, 5, 29]. This may suggest collapse of the primary health care system or lack of collaboration between the ministries of health and education in the study area. There is therefore, the need for all stakeholders to rise to this challenge.

Regarding teacher's preparation for health teaching, although most of the teachers claimed to have had trainings in components of SHI, Only 3.0% of them had engaged in In-service training in the last five years preceding this study compared to an average of between 35 and 65% recorded for teachers in Kenya a fellow African country [30]. This is disturbing bearing in mind the identified deficiencies in the training of primary school teachers in Nigeria [9] with some of them lacking adequate health knowledge [10, 11, 23, 24]. In-service training could be used to remedy deficiencies in the training of the teachers and update them with recent developments on school health issues given the probability that many of them might have graduated for long with the possibility of recall bias or failure. Stakeholders in the study area could make use of support programmes for improving teachers trainings and competence such as The United Nations Education, Scientific and Cultural Organisation‘s (UNESCO) Teachers‘ Training Initiative for Sub-Saharan Africa (TTISSA): A ten year programme launched in 2006 to improve the quality of teachers‘ training and status in Africa among other functions [30]. The call for action now cannot be more apt going by the words of UNESCO‘s Director General Dr. Irina Bokova at the commemoration of year 2013 teachers‘ day that “Teachers‘ professional knowledge and skills are the most important factors for quality education. This World Teachers‘ Day, we call for teachers to receive stronger training upfront and continual professional development and supports” [31]. This training will serve the dual purpose of enhancing their proficiency and motivation.

That none of the schools attained up to the minimum acceptable score in the aspects of school health instruction calls for concern and is a reflection that no meaningful health instruction was being given in the study area. This sharply contrasts with what is obtainable in the developed world [8]. With the revelation by the Centre for Disease Control USA that, health education is a cost effective way of meeting health needs of a society [7] and the captive nature of primary schools as avenues to positively modify the health behaviours of a country citizens through effective use of SHI on school children [28, 32, 33], given that the right methods are used [27, 28, 34, 35]. It is evident that, appropriately implemented SHI may be a cost effective way of meeting the health and education related MDGS for a developing nation like Nigeria.

Feedbacks from school based health instruction interventions [27, 28, 32–36] resulted in both teachers and school children having better knowledge on health matters with positive attitudes towards implementing school health instruction by the teachers. This corroborate the belief that, given the right training, environment and motivation, teachers and school children can serve as agents of change at reviving the poor state of health in their various communities.

## Conclusion

This study has shown a lot of deficiencies in both human and material resources needed for the provision of appropriate SHI in the study area. Inadequacies in SHI teaching time allocation, SHI curriculum contents delivery, In-service training of teachers and instructional materials were observed. Some of these factors have been adduced for the poor implementation of the UBE in Nigeria [7, 17]. This suggests some form of harmful synergy and might be an indication that effective SHI is missing in the study area. The relative high presence of PTA in this study could be explored for funding of programmes targeted at improving SHI in the area as a way of enhancing community participation in it‘s effective delivery. Provision of appropriate teachers‘ training, adequate instructional materials and government funding with proper oversight functions by relevant authorities will go a long way in helping the schools to attain effective SHI. There is need for further research geared towards ascertaining the practice, relevance, appropriateness and feasibility of implementing the current health curriculum in Nigerian primary schools as some authors‘ have hinted of the possibility of it‘s being over bloated [4].

## References

[1] Akani NA, Nkanginieme KEO, Azubike JC, Nkanginieme KEO (2007). School health programme. Paediatric and Child Health in a tropical region?.

[2] Ogundele BO, Ademuwagun ZA, Ajala JA, Oke EA, Moronkola OA, Jegede AS (2002). School health education. Health Education and Health promotion.

[3] Implementation Guidelines on National School Health Programme Federal Ministry of Education Nigeria 2006 Document. http://www.unicef.org/nigeria/sch_health_policy.pdf.

[4] Emechebe SN (2012). Achieving Universal Basic Education in Nigeria: Issues of relevance, quality and efficiency. Glob, Voice Edu..

[5] Akani NA, Nkanginieme KEO, Oruamabo RS (2001). The School health programme: A situational revisit. Nig J Paediatr.

[6] World Bank Documents Country and Lending groups. http://www.worldbank.org/countryandlendinggroups.htm.

[7] A guide for Florida's school health advisory committees: utilizing coordinate school health approach. http://www.doh.state.flus/family/school/coordinate/coordinated.html.

[8] Kann L, Telljohann SK, Wooley SF (2007). Health education: Results from the School Health Policies and Programme study 2006. J Sch Health.

[9] Agusiegbe GO (1988). Teacher preparation for health teaching in primary schools: primary health care strategy for all in the year 2000. Nig Schl Hlth J..

[10] Akani NA, Nkanginieme KEO, Oruamabo RS (2000). An evaluation of health knowledge of headteachers in Obio-Akpor primary schools and the effect of short term training on this knowledge Benin. J Edu Std..

[11] Alex- Hart BA, Akani NA, Nkanginieme KEO (2010). Evaluation of the health knowledge of teachers in public primary schools in Bonny local government area of Rivers state. Port Harcourt med J..

[12] (2009). Legal notice on publication of 2006 census final results of the Federal Republic of Nigeria, 2006 National Population Census Official Gazette. Annexure A,B and C.

[13] Anderson CL, Creswell WH (1980). School health practice St Louis: The CV Mosby company. Nig J Paediatr..

[14] Nigerian Educational Research and Development Council (NERC) (2007). The 9-year basic education curriculum; physical and health education for primaries 1-3 and 4-6. http://www.placng.org/lawsofnigeria/node/121.

[15] (2004). National Policy On Education of the Federal Republic of Nigeria. Federal Ministry of Education.

[16] Nigeria Universal basic education. http://www.ubeconline.com.

[17] Popoola Labo SO, Bello AA, Atanda FA (2009). Universal Basic Education In Nigeria: challenges and way forward. The Soc Sci..

[18] Joseph K (2007). Nigeria reproductive health, child health and education household, school and health facility midline surveys USAID reports October. http://www.cpc.unc.edu/measure.

[19] United Nations International Children's Emergency Fund (UNICEF). State of The World's Children (SOWC).

[20] Millenium Development Goals. http://www.un.org/milleniumgoals.

[21] Klakk HK, Chanipaw M, Heidemann M, Andersen LB, Wedderkopp N (2013). Effect of four additional physical education lessons on body composition in children aged 8-13 years. BMC Pediatr.

[22] Olaleye O, Adeyemo A, Ogunmade O, Opiah A, Okoh N The untamed business of kidnapping in Nigeria. http://www.thisdaylive.com.

[23] Ofovwe GE, Ofili AN (2007). Knowledge, attitude and practice of school health programme among head teachers of primary schools in Ego Local Government Area of Edo State, Nigeria. Ann Afr Med..

[24] Akpan MU, Ikpeme EE, Utuk E (2013). Teachers knowledge and attitudes towards seizure disorder: A comparative study of urban and rural school teachers in Akwa Ibom State. Niger J Clin Pract..

[25] Nwachukwu AE (2004). Implementation of school health programme in the past and present in Imo State Nigeria. Nig Schl Hlth J..

[26] Idehen CO, Oshodin OG (2008). Factors affecting health instruction in secondary schools in Edo state. Ethno med..

[27] Onyango-Ouma W, Aagaard-Hansen J, Jensen BB (2004). Changing concepts of health and illness among children of primary school age in Western Kenya. Health Educ. Res..

[28] Onyango-Ouma W, Aagaard-Hansen J, Jensen BB (2005). The potential of school children as health change agents in rural Western Kenya. Soc Sci Med..

[29] Morley D, Joseph MO, Nnanda WP, Gurage TH, Karine E, Kartz F Teachers and pupils as health workers in: mobilizing education to reinforce primary health care notes, comments (child, family, community). Digest No X.

[30] United Nations Educational Scientific and Cultural Organisation (UNESCO) Institute for Statistics. Teachers and educational Quality monitoring global needs for 2015.

[31] Rina B (2013). World Teachers?. Day speech.

[32] Ayi I, Nonaka D, Adjovu JK, Hanafusa S, Jimba M, Bosompem KM, Mizoue KMT, Takeuch T, Boakye DA, Kobayashi J (2010). School-based participatory health education for malaria control in Ghana: engaging children as health messengers. Malar J..

[33] Dzikwi AA, Ibrahim AS, Umoh JU (2012). Knowledge, attitude and practice about rabies among children receiving formal and informal education in Samaru, Zaria, Nigeria. Glob J Health Sci..

[34] Sufiyan MB, Sabitu KK, Mande AT (2012). Evaluation of the effectiveness of deworming and participatory hygiene education strategy in controlling anaemia among children aged 6 -15 years in Gadagau community, Giwa LGA, Kaduna, Nigeria. Ann Afr Med..

[35] Xuan le TT, Rheinlander T, Hoat LN, Dalsgaard A, Konradsen F (2013). Teaching handwashing with soap for schoolchildren in a multi-ethnic population in northern rural Vietnam. Glob Health Action.

[36] Xuan LT, Hoat LN, Rheinlander T, Dalsgaard A, Konradsen F (2012). Sanitation behaviour among school children in a muliti- ethnic area of Northern rural Vietnam. BMC Public Health.

